# Factors associated with quality of life in patients receiving lung transplantation: a cross-sectional study

**DOI:** 10.1186/s12890-023-02526-0

**Published:** 2023-06-23

**Authors:** Ryo Takahashi, Tamao Takahashi, Yoshinori Okada, Masahiro Kohzuki, Satoru Ebihara

**Affiliations:** 1 Department of Internal Medicine and Rehabilitation Science, Tohoku University Graduate School of Medicine, 1-1, Seiryo-Machi, Aoba-Ku, Sendai, Japan; 2 Department of Thoracic Surgery, Institute of Development, Aging and Cancer, Tohoku University, Sendai, Japan; 3 Yamagata Prefectural University of Health Sciences, Yamagata, Japan

**Keywords:** Respiration Disorders, Lung Transplantation, Quality of Life, Respiratory Function Tests, Exercise Tolerance, Dyspnea, Rehabilitation

## Abstract

**Background:**

With improved prognosis after lung transplantation (LTx), improving health-related quality of life (HRQL) in patients who have undergone LTx is a key goal. Although HRQL is improved significantly after transplantation, it is poorer than that in ordinary healthy people. However, the factors associated with poor HRQL remain unclear. This cross-sectional study aimed to identify the factors associated with poor HRQL in patients who have undergone LTx.

**Methods:**

Between December 2018 and May 2022, 80 patients who had undergone LTx completed St. George’s Respiratory Questionnaire (SGRQ) as a disease-specific quality of life measure, the Short Form-12 (SF-12) as a generic quality of life measure, and modified Medical Research Council (mMRC) scale of dyspnea. The groups were assigned according to the median SGRQ-total score and the Japanese population standard for SF-12, and those with good HRQL were compared with those with poor HRQL. Independent factors were evaluated using multivariate analysis.

**Results:**

With regard to the SGRQ, there were significant differences in the forced expiratory volume in 1 s (FEV_1_) (*P* = 0.041), use of bronchodilators (*P* = 0.026), 6-min walk distance (6MWD) (*P* < 0.001), and Mmrc (*P* < 0.001) between better and poorer HRQL. For the SF-12 physical component summary score (PCS), age (*P* = 0.017), sex (*P* = 0.011), FEV_1_ (*P* < 0.001), forced vital capacity (FVC) (*P* < 0.001), diagnosis (*P* = 0.011), handgrip force (*P* = 0.003), 6MWD (*P* < 0.001), and Mmrc (*P* < 0.001) varied. Multivariate analyses revealed that Mmrc was the only independent factor in the SGRQ (*P* < 0.001, odds ratio [OR] = 6.65, 95% confidence interval [CI]: 2.49–17.74) and SF-12 PCS (*P* = 0.001, OR = 0.185, 95% CI: 0.07–0.52). There were significant correlations between the SGRQ-Total score and SF-12 PCS (correlation coefficient = -0.612, *P* < 0.001).

**Conclusions:**

Dyspnea may be an independent factor of poor disease-specific and generic HRQL in LTx patients. The management of dyspnea may improve the HRQL in patients who have undergone LTx.

## Background

Lung transplantation (LTx) is one of the most effective ways of treating terminal lung diseases. The clinical aim of LTx is to improve the survival rate and Health-Related Quality of Life (HRQL) [1]. The post-transplant survival rate of LTx has improved over time [2]. A 5-year unadjusted survival rate of 54% has been reported by the International Society for Heart and Lung Registry [3]. At our institute, the five-year survival rate is around 75% [4]. With the improved prognosis after LTx, the importance of improving HRQL in patients has been recognized [5, 6].

Previous reports have shown a significant improvement in HRQL in patients 3–6 months after transplantation compared with that before transplantation [6, 7]. However, these reports have also shown that HRQL in patients who have undergone LTx was poorer than that in ordinary healthy people. Some reports have indicated that chronic lung allograft dysfunction (CLAD) is a possible factor leading to poor HRQL [5, 8, 9]. Although CLAD is defined as a decrease in forced expiratory volume in 1 s (FEV_1_) [10], there may be a dissociation between the results of lung function tests and the patients’ perception of their own health status or disease. Some patients who show a decrease in FEV_1_ do not report poor health status or exercise tolerance [11]. At present, the factors associated with poor HRQL remain unclear.

Instruments for measuring HRQL are broadly categorized as disease-specific or generic. The advantages of one category typically offset the limitations of the other. Generic HRQL does not reflect respiratory disease-specific symptoms such as dyspnea, cough, and wheezing. Therefore, it is common to utilize both disease-specific and generic instruments in HRQL assessment [11]. In this study, HRQL was evaluated using both disease-specific and generic HRQL measures.

This study aimed to identify the independent factors for poor HRQL in patients after LTx by comparing patients who have better HRQL with those who have poor HRQL in both disease-specific and generic aspects of HRQL. The results may also lead to new therapies to improve HRQL in patients who have undergone LTx with long-term survival.

## Methods

### Participants and study design

Between March 2000 and May 2021, 136 patients underwent LTx at the Tohoku University Hospital. Among these patients, 91 patients aged ≥ 18 years who had undergone LTx > 3 months prior and were regularly scheduled for clinical follow-up visits at our hospital were included in this cross-sectional study. Forty-five patients were excluded due to the following reasons: four were aged < 18 years, three were admitted to the ward due to deterioration of lung function, and 38 died. Eight patients declined participation or were too disabled to meet. Written informed consent was obtained from all 83 patients. They completed HRQL questionnaires by themselves during their follow-up visit to our hospital between December 2019 and December 2021. Three patients were excluded from the analysis because of missing data. Thus, 80 patients were included in the final analysis. This study was approved by the Medical Ethics Committee of Tohoku University Hospital (approval number: 20181432).

### Health-related quality of life

The St. George’s Respiratory Questionnaire (SGRQ) was used to evaluate respiratory disease-specific HRQL [12], whereas the Medical Outcomes Survey Short Form-12 version 2 in Japanese (SF-12) was used to evaluate generic HRQL [13].

The SGRQ is a respiratory disease-specific instrument designed to measure HRQL for chronic obstructive pulmonary disease (COPD) that has been used recently to measure HRQL for various respiratory diseases, such as lymphangioleiomyomatosis (LAM) [14] and idiopathic interstitial pneumonia [15]. An HRQL evaluation method specific to patients who have undergone LTx has not yet been fully established. The most frequently employed respiratory disease-specific HRQL instrument in patients who have undergone LTx is the SGRQ [5, 8, 16–18]. The scores range from 0 to 100, with higher scores indicating poorer health. The SGRQ has three components: symptoms, activities, and impacts. The total score was calculated from the summation of these three components.

The SF-12 is a validated and reliable instrument adapted from the Short Form 36-Item Health Survey that has a mean of 50 and standard deviation of 10 for the general Japanese population [19]. The SF-12 comprises the Physical Component Summary Score (PCS) and Mental Component Summary Score, which are widely used to evaluate generic HRQL across various populations [7, 20, 21]. Each scale is scored from 0 to 100, with higher scores indicating better HRQL.

The patients were allocated into two groups based on the median SGRQ-Total score. The group with a score higher than the median score of the SGRQ-Total score for all patients was designated as the high-SGQR (poor respiratory-specific HRQL) group, and the group with a lower score was designated as the low-SGQR (better respiratory-specific HRQL) group. The patients were allocated into two groups according to the Japanese population norm in the SF-12 PCS. The group with a higher score than 50 was designated as the high-PCS (better generic HRQL) group, and the group with a lower score was designated as the low-PCS (poor generic HRQL) group.

### Other measures

The modified Medical Research Council (mMRC) scale was used to assess dyspnea. mMRC is a unidimensional five-point scale that describes almost the entire range of respiratory disability, from none (Grade 0) to almost complete incapacity (Grade 4). Higher scores indicate worse dyspnea [22].

Clinical data regarding, including age, sex, body mass index (BMI), time since LTx, lung function test, diagnosis before LTx, type of transplant, CLAD, hospitalization for pulmonary infections, home oxygen therapy (HOT), use of bronchodilators, use of anti-fibrotic agents, handgrip strength assessed using peak handgrip force on the dominant hand with a dynamometer, and 6-min walking distance (6MWD), were obtained from the medical records.

The same immunosuppressive protocol was implemented for all patients after LTx at our institute. All patients received 10–14 ng/ml of tacrolimus for the first 6 months, followed by 9–13 ng/ml until 12 months and 8–10 ng/ml thereafter. In addition, patients weighing above 50 kg received 1500 mg of mycophenolate, whereas those weighing below 50 kg received 1000 mg of mycophenolate. The patients also received 1.0 mg/kg of prednisolone for the first 4 days, and the dose was gradually decreased to 5 mg thereafter [4]. Patients who could not tolerate tacrolimus or mycophenolate received cyclosporine or azathioprine, respectively. None of the patients received anti-fibrotic agents during the follow-up period.

### Statistical analysis

Continuous variables are presented as the mean and standard deviation, and categorical variables are presented as count frequencies. The Shapiro–Wilk test was used to compare the distributions with the standard normal distribution.

Non-normally distributed variables were compared using the Mann–Whitney U test, and normally distributed variables were compared using the t-test. Associations between categorical variables were estimated using the chi-squared test. Statistical significance was set at *P*-value < 0.05. Variables with *P*-values < 0.1 on comparison between the groups were subsequently analyzed using multivariate logistic regression to identify the independent factors of poor HRQL. Analyses were performed using the statistical software package IBM SPSS version 26.0 (SPSS Inc., Chicago, IL, USA).

## Results

**Fig.1:**
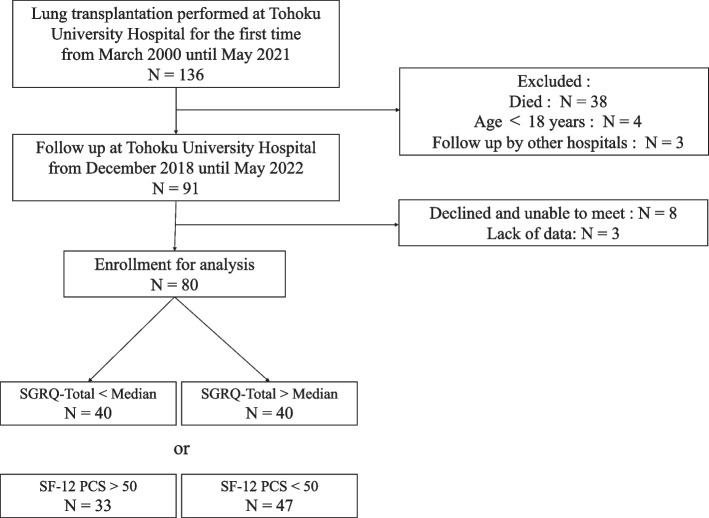
Flow diagram of the participants

The flow diagram of the patients is shown in Fig. 1. The clinical characteristics of the study participants are shown in Table 1. Among the 80 participants, 48 (60%) were female, the average age was 47.9 ± 10.7 years, and the average time since LTx was 5.6 ± 4.3 years. The average FEV_1_ was 1.85 ± 0.69 L, and the average %FEV_1_ was 63.1 ± 19.1% predicted. Regarding diagnoses, 26 (32.5%) patients were diagnosed with LAM, 19 (23.8%) patients were diagnosed with pulmonary hypertension (PAH), 18 (22.5%) patients were diagnosed with interstitial pneumonia (IP), and seven (8.8%) patients were diagnosed with COPD. CLAD was diagnosed in 13 patients. The use of bronchodilators was reported in 23 patients, whereas the use of antifibrotic agents was not reported in any patient. The mean 6MWD was 504 ± 89.3 m. Regarding mMRC, 20 (25.0%) patients had Grade 0, 30 (37.5%) patients had Grade 1, 27 (33.8%) patients had Grade 2, and three (3.8%) patients had Grade 3.

**Table 1:** Patient characteristics (*N* = 80)

Variables	
Age, y	47.9 ± 10.7
Sex, female/male, n	48/32
Time since LTx, y	5.6 ± 4.3
Body mass index, kg/m^2^	20.0 ± 4.3
FEV_1_, L	1.85 ± 0.69
FEV_1_, % predicted	63.1 ± 19.1
FVC, L	2.41 ± 0.75
FVC, % predicted	69.2 ± 19.4
FEV_1_/FVC	77.7 ± 18.6
FEV_1_/FVC, % predicted	91.9 ± 20.6
Diagnosis, *n*	
LAM	26
PAH	19
IP	18
COPD	7
Others	10
Type of LTx, n	
Brain-dead-donor single	46
Brain-dead-donor double	29
Living-donor double	5
CLAD, n	13
Hospitalization for pulmonary infections, n	29
HOT, n	8
Bronchodilator use, n	23
Handgrip force, kg	27.9 ± 7.9
6MWD, m	504 ± 89.3
mMRC dyspnea, n	
Grade 0	20
Grade 1	30
Grade 2	27
Grade 3	3
SGRQ	
Symptom	27.5 ± 20.2
Activity	44.6 ± 25.5
Impact	23.1 ± 18.1
Total	30.4 ± 18.8
SF-12	
PCS	46.3 ± 14.0
MCS	56.4 ± 7.3

Data are expressed as numbers or means ± SD

*LAM* lymphangioleiomyomatosis, *PAH* pulmonary arterial hypertension, *IP* interstitial pneumonia, *COPD* chronic obstructive pulmonary disease, *LTx* lung transplantation, *CLAD* chronic lung allograft dysfunction; HOT, home oxygen therapy, *6MWD* 6-min walk distance, *mMRC* modified medical research council dyspnea scale, *SGRQ* St. George's respiratory questionnaire, *SF-12* medical outcomes survey short form-12, *PCS* physical component summary score, *MCS* mental component summary score; SD, standard deviation

The characteristics of the high-SGRQ and low-SGRQ groups are shown in Table 2. There were significant differences in FEV_1_, use of bronchodilators, 6MWD, and mMRC between the groups. There were no differences in age, sex, FVC, diagnosis, CLAD, hospitalization for pulmonary infections, or handgrip force between the groups.

**Table 2:** Comparison of the low- and high-SGRQ groups

Variables	Low-SGRQ *N* = 40	High-SGRQ *N* = 40	*P* value
Age, y	46.4 ± 10.3	49.4 ± 11.7	0.25^†^
Sex, female/male, n	21/19	27/13	0.50^‡^
Time since LTx, y	5.7 ± 4.0	5.4 ± 5.1	0.45^*^
Body mass index, kg/m^2^	20.8 ± 4.7	19.2 ± 3.7	0.13^*^
FEV_1_, L	2.00 ± 0.72	1.70 ± 0.64	0.041^*^
FEV_1_,% predicted	65.7 ± 18.8	60.1 ± 19.2	0.34^*^
FVC, L	2.50 ± 0.70	2.3 ± 0.80	0.40^†^
FVC, % predicted	69.9 ± 19.2	68.5 ± 19.7	0.67^*^
FEV_1_/FVC, %	80.0 ± 15.6	75.3 ± 21.1	0.26^*^
FEV_1_/FVC, % predicted	92.8 ± 19.1	90.6 ± 22.3	0.83^*^
Diagnosis, n			0.62^‡^
LAM	11	15	
PAH	10	9	
IP	8	10	
COPD	4	3	
Others	7	3	
Type of LTx, n			0.26^‡^
Brain-dead-donor double	18	11	
Brain-dead-donor single	20	26	
Living-donor double	2	3	
CLAD, n	8	5	0.55^‡^
Hospitalization for pulmonary infections, n	14	15	0.82^‡^
HOT, n	2	6	0.26^‡^
Bronchodilator use, n	7	16	0.026^‡^
Handgrip force, kg	29.3 ± 7.3	26.5 ± 8.2	0.057^*^
6MWD, m	536 ± 83.6	473 ± 84.4	< 0.001^*^
mMRC, n			< 0.001^‡^
Grade 0	17	3	
Grade 1	20	10	
Grade 2	3	24	
Grade 3	0	3	

Data are expressed as numbers or means ± SD

*LAM* lymphangioleiomyomatosis, *PAH* pulmonary arterial hypertension, *IP* interstitial pneumonia, *COPD* chronic obstructive pulmonary disease, *LTx* lung transplantation, *CLAD* chronic lung allograft dysfunction, *HOT* home oxygen therapy, *6MWD* 6-min walk distance, *mMRC* modified medical research council dyspnea scale, *SD* standard deviation

^*^Mann–Whitney U-test

^†^Student t test

^‡^Chi-squared test

Multiple regression analysis was performed to identify the independent factors for the SGRQ. In the SGRQ, variables with *P*-values < 0.1 were FEV_1_, handgrip strength, 6 MWD, and mMRC. Multivariate analysis revealed that only mMRC (*P* < 0.001, odds ratio [OR] = 6.65, 95% confidence interval [CI]: 2.49–17.74) was independently associated with the SGRQ (Table 3).

**Table 3:** Multivariate logistic regression analysis of the predictors of the SGRQ-total score

	Odds ratio	95% CI	*P* value
Age, y	1.002	0.95–1.06	0.95
Sex, female	0.712	0.07–7.18	0.77
FEV_1_, L	1.265	0.42–3.79	0.67
Bronchodilators use, n	1.723	0.42–7.05	0.45
Handgrip force, kg	0.958	0.83–1.10	0.54
6MWD, m	0.998	0.99–1.01	0.57
mMRC	6.649	2.49–17.74	< 0.001

*FEV*_*1*_ forced expiratory volume in 1 s, *6MWD* 6-min walk distance, *mMRC* modified medical research council dyspnea scale

The characteristics of the high- and low-PCS groups are shown in Table 4. The number of participants in the high-PCS group was smaller than that in the low-PCS group. There were significant differences in age, sex, FEV_1_, FVC, diagnosis (COPD), handgrip force, 6 MWD, and mMRC between the groups. There were no differences in CLAD or hospitalization for pulmonary infections between the groups. Multiple regression analysis was performed to identify the independent factors for the SF-12 PCS. In the SF-12 PCS, variables with *P-*values < 0.1 were, age, sex, FEV_1_, handgrip strength, 6 MWD, and mMRC. The analysis revealed that only mMRC (*P* = 0.001, OR = 0.185, 95% CI: 0.07–0.52) was independently associated with the SF-12 PCS (Table 5).

**Table 4:** Comparison of the high- and low-PCS groups

Variables	High-PCS *N* = 33	Low-PCS *N* = 47	*P* value
Age, y	44.0 ± 10.7	50.6 ± 9.9	0.017^*^
Sex, female/male, n	14/19	34/13	0.011^‡^
Time since LTx, y	4.8 ± 3.9	6.06 ± 4.9	0.33^*^
Body mass index, kg/m^2^	20.1 ± 3.3	19.9 ± 4.9	0.36^*^
FEV_1_, L	2.14 ± 0.75	1.64 ± 0.57	< 0.001^†^
FEV_1_, % predicted	67.1 ± 19.7	60.3 ± 18.3	0.12^†^
FVC, L	2.74 ± 0.76	2.18 ± 0.65	< 0.001^†^
FVC, % predicted	78.2 ± 22.1	66.9 ± 17.1	0.21^†^
FEV_1_/FVC, %	77.3 ± 19.8	78.2 ± 16.9	0.83^*^
FEV1/FVC, % predicted	91.7 ± 21.2	92.1 ± 20.1	0.98^*^
Diagnosis, n			0.011^‡^
LAM	7	19	
PAH	11	8	
IP	4	14	
COPD	6	1	
Others	5	5	
Type of LTx, n			0.13^‡^
Brain-dead-donor double	16	13	
Brain-dead-donor single	16	30	
Living-donor double	1	4	
CLAD, n	3	10	0.22^‡^
Hospitalization for pulmonary infections, n	11	18	0.65^‡^
HOT, n	1	7	0.08^‡^
Bronchodilator use, n	7	16	0.21^‡^
Handgrip force, kg	30.7 ± 7.4	25.9 ± 7.6	0.003^*^
6MWD, m	551 ± 79.9	472 ± 81.4	< .001^*^
mMRC, n			< .001^‡^
Grade 0	16	4	
Grade 1	14	16	
Grade 2	3	24	
Grade 3	0	3	

Data are expressed as numbers or means ± SD

*FEV*_*1*_ forced expiratory volume in 1 s, *FVC* forced vital capacity, *LAM* lymphangioleiomyomatosis, *PAH* pulmonary arterial hypertension, *IP* interstitial pneumonia, *COPD* chronic obstructive pulmonary disease, *LTx* lung transplantation, *CLAD* chronic lung allograft dysfunction, *HOT* home oxygen therapy, *6MWD* 6-min walk distance, *mMRC* modified medical research council dyspnea scale, *SD* standard deviation

^*^Mann–Whitney U test

^†^Student t test

^‡^Chi-squared test

**Table 5:** Multivariate logistic regression analysis of the predictors of SF-12 PCS

	Odds ratio	95% CI	*P* value
Age, y	0.948	0.89–1.01	0.08
Sex, female	1.228	0.11–14.20	0.87
FEV_1_, L	1.179	0.28–4.92	0.82
FVC, L	1.810	0.45–7.25	0.40
COPD	8.653	0.53–141.67	0.13
HOT	0.747	0.03–20.00	0.86
Handgrip force, kg	1.010	0.87–1.17	0.90
6MWD, m	1.003	0.99–1.01	0.50
mMRC	0.185	0.07–0.52	0.001

*FEV*_*1*_ forced expiratory volume in 1 s, *FVC* forced vital capacity, *6MWD* 6-min walk distance, *COPD* chronic obstructive pulmonary disease, *HOT* home oxygen therapy, *mMRC* modified medical research council dyspnea scale

**Fig. 2:**
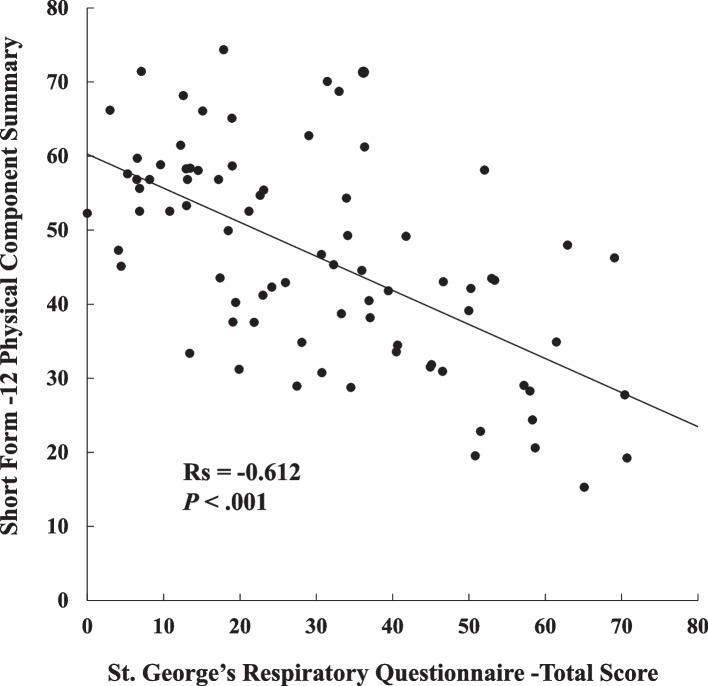
Correlation between the St. George’s respiratory questionnaire-total score and the short form-12 physical component summary score

There was a significant correlation between the SGRQ-Total Score and SF-12 PCS (Spearman correlation coefficient Rs = -0.612, *P* < 0.001) (Fig. 2).

## Discussion

This study aimed to elucidate the independent factors that had the greatest influence on HRQL in patients who have undergone LTx. The mean SGRQ-Total score in our study was consistent with that reported in previous studies [8, 18, 23]. The mean SF-12 PCS in our study was also consistent with that reported in previous studies [7].

Previous studies have shown that at about 3 months after transplantation, patients experienced significant improvement in the SF-12 PCS, but no significant improvement was observed between 6 and 36 months [7, 24]. Despite substantial improvements in the generic HRQL, these patients could not reach the population norm in PCS [5, 7, 9]. Physical functioning is a particularly important factor for the improvement of HRQL, as the greatest improvements in HRQL are achieved after the improvement of physical functioning following LTx [11].

In our study, there were significant differences in FEV_1_, 6MWD, and mMRC between the Low-SGRQ and High-SGRQ groups. There was no significant difference in the incidence of CLAD between the low-SGRQ and high-SGRQ groups. Previous studies have shown that respiratory-specific HRQL is also worse in patients with CLAD compared with that in those without CLAD [5, 8]. Patients with advanced CLAD had profound dyspnea [11]. Severe CLAD can affect HRQL if dyspnea emerges as a subjective symptom. The lack of a significant difference related to CLAD in our data can be attributed to the small number of CLAD cases.

The multivariate logistic regression model revealed that only mMRC was an independent risk factor significantly associated with the SGRQ (Table 3). These results suggest that the risk factor that affects disease-specific HRQL is a subjective symptom rather than objective factors, such as lung function and exercise tolerance.

There were differences in age, sex, FEV_1_, FVC, diagnosis, handgrip force, 6 MWD, and mMRC between the Low-PCS and High-PCS groups. The ages of the patients in our study ranged from 22 to 70 years. Since it has been reported that quality of life due to physical function declines with age [11, 19], age differences in our results might lead to differential physical activity levels, resulting in differences in generic HRQL. In our study, the high proportion of females was attributed to LAM, which affects only women, being the most frequently diagnosed disease before LTx. A previous study reported poorer HRQL in women than that in men. Shahabeddin et al. [25] reported that poor HRQL among females was related to the decline in the physical domains of the Nottingham Health Profile, a generic HRQL. In our study, there was a difference in the type of disease between the low- and high-PCS groups, especially COPD. Since six of the seven patients with COPD were males, COPD may have contributed to the higher PCS.

Although Künsebeck et al. reported that the SF-12 was negatively affected by the onset of severe CLAD [20], the incidence of CLAD did not affect the SF-12 in our study. Similar to the SGRQ, this is because the number of cases of CLAD in our data was small.

The multivariate logistic regression model revealed that only mMRC was significantly associated with the SF-12 PCS scores (Table 5). The independent factor associated with better SF-12 PCS was lower dyspnea sensation, which is a patient-reported outcome, suggesting that subjective symptoms influenced generic HRQL more than objective indicators such as lung function tests and exercise tolerance.

The severity of dyspnea as a subjective symptom, rather than lung function and exercise tolerance, could be an independent factor that lowers HRQL in patients who have undergone LTx. There is a correlation between the SGRQ-Total score as a disease-specific HRQL measure and the SF-12 PCS as a generic HRQL measure (Fig. 2), suggesting that the management of dyspnea plays a pivotal role in improving the quality of life of patients who have undergone LTx.

Dyspnea is a multidimensional symptom. A comprehensive multidisciplinary approach to respiratory rehabilitation, including physical, emotional, social, and spiritual aspects of refractory dyspnea, is important according to the symptoms and background of each patient [26].

Some studies have shown that comprehensive respiratory rehabilitation, which is a combination of exercise training and education, can improve exercise tolerability and HRQL in pulmonary disease [27, 28]. Fuller et al. concluded that 7 and 14 weeks of supervised training after LTx improved exercise tolerance, muscle strength, and the SF-36 PCS scores at 14 weeks, with no significant differences between the two groups [29].

Exertional dyspnea is consistently reported to be reduced by pulmonary rehabilitation [30]. For example, in a report on patients with chronic dyspnea, HRQL and life expectancy were significantly improved by eliminating the cause of dyspnea, physical therapy, occupational therapy, the introduction of social services by social workers, and palliative and spiritual approaches [31]. Exertional dyspnea is an important problem for some patients whose symptoms worsen over time after LTx. Management of dyspnea is also important in patients who do not recover well after LTx. Continued comprehensive respiratory rehabilitation tailored to dyspnea may improve HRQL long after LTx. However, few long-term follow-up studies have been conducted on comprehensive respiratory rehabilitation after LTx. Thus, further research is required in the near future.

Our study had several limitations. First, the survivor bias in HRQL evaluation in patients with relatively good prognoses after LTx was a major limitation. Second, cross-sectional analysis has an inherent limitation in interpreting causes and effects. Although this study examined postoperative HRQL, preoperative respiratory rehabilitation has been shown to improve HRQL [32]. Further studies are needed to compare preoperative and postoperative HRQL in patients who have undergone LTx and are candidates for it.

## Conclusions

Dyspnea is an independent factor that reduces both disease-specific and generic HRQL in patients who have undergone LTx. The management of dyspnea may play a pivotal role in improving HRQL in patients who have undergone LTx.

## Data Availability

The datasets used and/or analyzed during the current study are available from the corresponding author on reasonable request.

## Abbreviations

BMI: Body mass index
COPD: Chronic obstructive pulmonary disease
CLAD: Chronic lung allograft dysfunction
FEV_1_: Forced expiratory volume in 1 s
FVC: Forced vital capacity
HOT: Home oxygen therapy
HRQL: Health-related quality of life
IP: Interstitial pneumonia
LAM: Lymphangioleiomyomatosis
MCS: Mental component summary
mMRC: Modified medical research council dyspnea scale
PCS: Physical component summary
PAH: Pulmonary arterial hypertension
PRO: Patient-reported outcome
SGRQ: St. George’s respiratory questionnaire
SF-12: Medical outcomes survey short form-12 version 2
6MWD: 6-Min walking distance
